# AI-Enabled Smart Glasses for Active Aging: Scoping Review

**DOI:** 10.2196/81157

**Published:** 2026-02-25

**Authors:** Claudio Delgado-Morales, Juan Jesús García-Iglesias, Ana Duarte-Hueros

**Affiliations:** 1 Department of Education, Universidad de Huelva, Avenida de las Fuerzas Armadas, Huelva, 21007, Spain, 34 626-206-343, 959219287; 2 Department of Sociology, Social Work and Public Health, Universidad de Huelva, Huelva, Spain

**Keywords:** smart glasses, artificial intelligence, AI, older adults, well-being, active aging

## Abstract

**Background:**

The daily use of digital technologies is transforming the day-to-day lives of older adults. Among these technologies, artificial intelligence–enabled smart glasses have recently emerged, which allow constant interaction with the device itself and with the environment. They are designed to be used for multitasking, including options such as being able to take photographs and/or videos; record immersive audio; make calls; and share multimedia content through voice commands, touch, or blink detection.

**Objective:**

The aim of this study was to map the existing evidence and gain insight into the effectiveness and potential benefits of artificial intelligence–enabled smart glasses in promoting active living in old age.

**Methods:**

A scoping review was performed following the PRISMA-ScR (Preferred Reporting Items for Systematic Reviews and Meta-Analyses extension for Scoping Reviews) statement by consulting the PubMed, Scopus, and Web of Science databases using a search strategy and syntax (“smart glasses” AND (“older adult” OR elderly OR aging) AND health). The review process was conducted through the Covidence online platform, and the final review protocol was prospectively registered in the Open Science Framework. Both the research question and eligibility criteria were based on the population, concept, and context framework for scoping reviews.

**Results:**

From a total of 58 papers identified, 6 (10.3%) studies were finally included (2 pilot studies, 2 technological development studies with experimental validation of a prototype, 1 mixed methods feasibility trial, and 1 survey) published between 2015 and 2023 after eliminating duplications and screening titles, abstracts, and keywords. The results suggest that the use of artificial intelligence–enabled smart glasses may contribute to improving the quality of life, independence, autonomy, motor functions, and social interactions of older adults.

**Conclusions:**

Because of the novelty of this type of digital technology, there is very little research on this topic at present. Moreover, the adoption and implementation of artificial intelligence–enabled smart glasses are conditioned by hindering factors such as data protection, the high price, and the lack of compatibility with conventional prescription glasses, as well as the lack of evaluation of their effectiveness, usability, and acceptance.

## Introduction

The aging of populations worldwide raises important questions for health systems and social policies. In this context, the promotion of active aging, understood as older people’s ability to adapt and function in their daily environment [1-3], has become a priority to improve the quality of life of older adults in terms of health, participation, and safety. Given the decline in physical and cognitive functioning with advancing age, coupled with demographic shifts toward an increasingly aging society, it is essential to contribute to improving the well-being of this population [4]. Emerging digital technologies offer new opportunities to support these objectives [5]. These technologies, which include information and communications technologies, websites, cloud computing, big data, 3D printing, social networks, software, mobile devices, robots, and artificial intelligence (AI), enable immediate access to and transmission of information. Additionally, they enable communication in multiple formats over telecommunication networks anytime and anywhere, interconnection between devices, and automation [6-9].

AI-enabled smart glasses have recently stood out among these technologies [10]. These are wearable devices designed for multitasking use, integrating advanced technologies such as displays, cameras, internet connectivity, and AI to offer additional functions beyond those of traditional glasses (eg, take photos, record audio and immersive videos, listen to music, make and receive calls, and share multimedia content) [11-13]. These devices can be controlled by users through voice commands, touch, or physical inputs such as blink detection.

The most advanced AI-enabled smart glasses use this technology to process and analyze data in real time, enhancing the user experience by interacting with the environment in the most efficient and enriching way [13]. Due to their technological features, AI-enabled smart glasses promise to help older adults gain higher daily functional autonomy for active aging by providing feedback, personalized assistance, and training, such as in taking medication, getting dressed, learning to eat more healthful food, or exercising [14-16]. Moreover, this technology takes advantage of the fact that older adults often wear traditional glasses [17].

While the potential of AI-enabled smart glasses has been studied in the area of health training [18,19], at the professional level [12,20-22], and as an assistive technology to support functional diversity [13,23,24], as well as in younger populations [4,25], research on their use in active living in old age, as a training resource, and/or in the promotion of the well-being of older adults is scarce. For this reason, a scoping review was carried out as it is appropriate for identifying key concepts that underpin a given field of research, as well as clarifying the definitions and/or conceptual boundaries of a topic [26-29].

The main objectives of this study were to map the available evidence and identify gaps in the existing research, as well as to examine the effectiveness and possible benefits of AI-enabled smart glasses in promoting active aging in older adults. We explore their potential impact and provide an overview of them, as well as clarify key concepts related to their use in fundamental areas such as autonomy, well-being, and social participation, while also identifying barriers that affect both the implementation and adoption of these devices.

## Methods

### Study Design

A scoping review was performed in accordance with the PRISMA (Preferred Reporting Items for Systematic Reviews and Meta-Analyses) statement [30], consistent with the standards and the updated and extended checklist for scoping reviews, PRISMA-ScR (PRISMA extension for Scoping Reviews) [31]. Similarly, relevant recommendations to ensure the thoroughness and quality of the process were taken into account in the different phases and drafting of this scoping review [29].

The final review protocol was prospectively filed with the Open Science Framework on April 16, 2025 [32], and is publicly accessible.

### Databases and Search Strategies

In March 2025, a total of 3 databases of high scientific impact, relevance, completeness, and functionality for research were consulted, with one of them specific to the health field given the nature of the study: PubMed, Scopus, and Web of Science. Two phases were determined to establish the search strategy. First, as phase 1, an iterative premapping was carried out in the 3 databases, combining terms such as “artificial intelligence glasses,” “well-being,” and “benefits” with those referring to older adults (“older adult,” “elderly,” and “aging”) to unify the keywords used in the first studies found and, in this way, be able to establish a subsequent search strategy that was as operational as possible. Thus, a network of co-occurring keywords was generated using the data visualization and analysis software VOSviewer (version 1.6.20; Centre for Science and Technology Studies, Leiden University) from a total of 10 preliminary documents: 2 (20%) from PubMed, 6 (60%) from Scopus, and 2 (20%) from Web of Science.

Then, as phase 2, based on the keywords co-occurring in the preliminary documents, it was decided to use and combine the following terms with the Boolean operators “AND” and “OR” using the following search strategy: (“smart glasses” AND (“older adult” OR elderly OR aging) AND health). In this way, where appropriate, words were combined to reflect the syntax and search parameters common to all 3 databases. In addition, it should be noted that the number of records generated did not vary when truncation was used in the term “health*.”

### Selection Criteria and Participant Demographics

The research questions were formulated based on the stated objectives and according to the eligibility criteria, as follows: (1) what scientific evidence exists and what knowledge gaps can be identified on the use of AI-enabled smart glasses in promoting active aging in older adults? (2) What is the effectiveness of AI-enabled smart glasses and what potential benefits do they offer in the promotion of active aging in older adults? (3) What barriers or factors reported in the literature may hinder both the implementation and adoption of AI-enabled smart glasses in older adults for the promotion of active aging?

As shown in Table 1, the population, concept, and context framework was used in this scoping review. It is a structure that streamlines the approach and definition of inclusion criteria in scoping reviews [29].

**Table 1.** Details of the population, concept, and context (PCC) framework and eligibility criteria.

PCC domain	Description
Population	Older people, ie, those aged >65 years, following the definition of the World Health Organization [33], and older adults who live in nursing homes or residences
Concept	Use of AI^a^-enabled smart glasses by older adults and their effectiveness and possible benefits in the promotion of active aging; AI-enabled smart glasses similar to Ray-Ban Meta, Solos AirGo Vision, Halliday glasses, Microsoft HoloLens 2, Google Glass, or Epson Moverio BT-300, among others; and wearable assistive technology devices with built-in AI and/or AI-automated mechanisms for older adults
Context	Use of AI-enabled smart glasses in daily life; for self-management of health; for educational purposes; in improving cognitive aspects, autonomy, and quality of life; as a means of leisure for interactive entertainment and/or social connections in one’s own home, in residences, or in health care settings; and consideration of barriers to implementation and/or adoption, such as technological availability, levels of digital literacy, technological accessibility, and factors inherent to socioeconomic and cultural conditions

a AI: artificial intelligence.

As this is a particularly recent topic and the use of this type of technology is on the rise, it was decided not to consider any exclusion criteria related to the type of document, year, area, and language, among others, so as not to discard related studies. The only exclusion criterion taken into account was that the studies should not be focused on conventional augmented or virtual reality glasses without built-in AI and/or capabilities that use AI through their software.

### Data Collection Procedures

To approach the review process, the online platform Covidence (Veritas Health Innovation) was used, which facilitates and optimizes the conduct of this type of study (eg, import of references, screening of records, blind review, data extraction, and risk of bias assessment). Two reviewers independently and blindly carried out the entire review process on this platform based on the proposed eligibility criteria, justifying and recording the reasons for exclusion. Any discrepancies during the selection of studies were discussed and resolved through consensus.

Data extraction, focused on identifying, organizing, and compiling the most relevant information related to the main objective of this review, was carried out in an exhaustive manner, including all the findings reported in the selected studies, to guarantee a rigorous and coherent analysis in accordance with the available evidence. The extracted data included the following details: (1) author and year of publication, (2) title, (3) type of document, (4) country of origin, (5) objective, (6) methodology, (7) sample, (8) type of AI-enabled smart glasses used and/or analyzed, and (9) main results.

### Data Analysis

After finalizing the data extraction process, the key findings obtained from the selected studies were summarized and presented in an evidence synthesis table (Multimedia Appendix 1 [14-16,23,34,35]).

In addition to examining the effectiveness and potential benefits of this type of technology in active living in old age, some barriers were detected that affect both the implementation and the adoption of AI-enabled smart glasses in this sector of the population. Furthermore, the gaps found in scientific literature regarding interventions based on these digital technologies were also synthesized, leading to the elaboration of a detailed summary of the objectives and findings of this study.

## Results

### Inclusion of Studies

Of a total of 58 records, 12 (20.7%) duplicate documents were eliminated. Subsequently, the remaining 79.3% (46/58) of the results were screened by analyzing the titles, abstracts, and keywords. Of these 46 papers, 39 (84.8%) were excluded because they were not related to the topic under study, not focused on AI-enabled smart glasses, and/or not targeted at adults older than 65 years. Therefore, 7 studies were reviewed in full. Of the 7 studies analyzed in full, 1 paper was excluded because it was not aimed at older adults and did not consider age (>65 years) among the participants.

**Figure 1.**
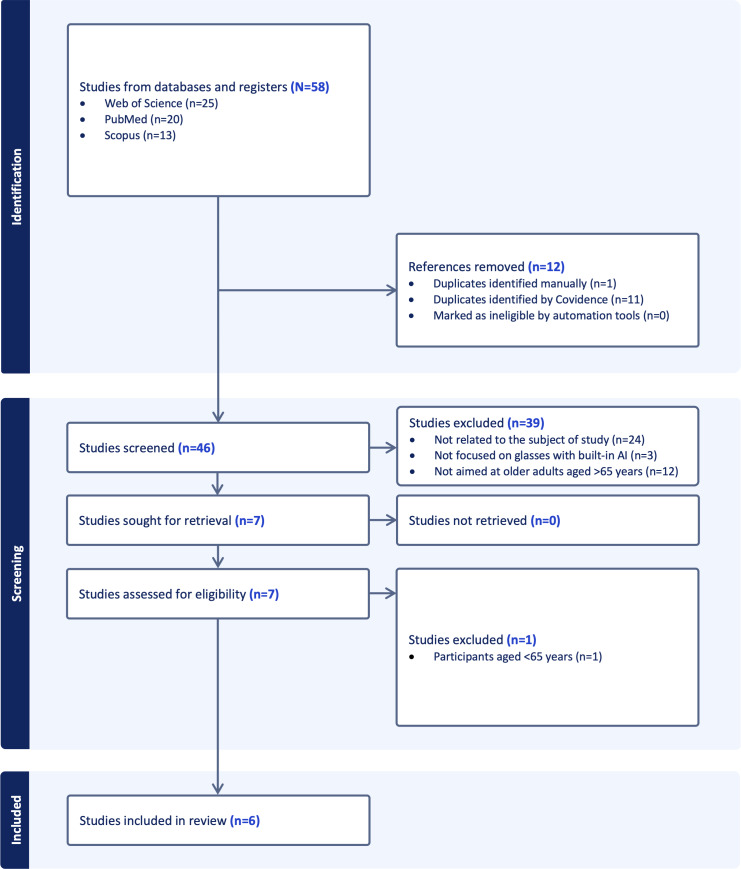
PRISMA-ScR (Preferred Reporting Items for Systematic Reviews and Meta-Analyses extension for Scoping Reviews) flowchart (extracted from Covidence). AI: artificial intelligence.

As shown in Figure 1 (flowchart adapted from the PRISMA-ScR guidelines) and Table 2, 6 studies were included in this scoping review.

**Table 2.** Summary of the included studies on artificial intelligence (AI)–enabled smart glasses for older adults.

Study	Sample	Glasses or device	Study design	Outcomes
Muema et al [14]	Older adults with cognitive impairment (not specified)	Microsoft HoloLens 2	Experimental integration study	Improved independence and autonomy via companion robot–AI-enabled smart glasses integration and enhanced emotion recognition
Tunur et al [15]	7 older adults with Parkinson disease	Google Glass	Mixed methods feasibility study	Safe and acceptable, improvements in motor function, and positive participant satisfaction
Zhao et al [16]	62 older adults with Parkinson disease	AI-enabled smart glasses (general)	Online survey	Positive attitudes toward adoption and perceived usefulness for daily activity support and self-confidence
Moshtael et al [23]	23 older adults with macular disease	Epson Moverio BT-200	Pilot quantitative study	Increased reading speed and satisfaction with personalized text display
Wang et al [34]	50 older adults	Epson Moverio BT-300	Experimental prototype study	Accurate facial recognition, improved text readability, and voice command control validated
Chang et al [35]	5 older adults	Jorjin J7EF AR smart glasses	Pilot quantitative study	Improved exercise adherence and recovery rates and AI-enabled smart glasses feasible for home-based fitness promotion

### Demographics of the Included Studies

Half of the papers were published in 2020 [15,23,34], with the publication period starting in 2015, when the first of the eligible studies was published [16], and including 2022 [14] and 2023 [35]. The studies were conducted in 5 different locations: Canada [14], the United States [15], the Netherlands [16], the United Kingdom [23], and Taiwan [34,35].

### Designs of Included Studies

Five of the 6 studies used a quantitative methodology [14,16,23,34,35], and 1 study adopted a mixed methods approach [15]. This was a single-group feasibility pilot test where previously validated questionnaires and standardized scales were administered, as well as semistructured interviews. Three studies used an experimental-technological perspective [14,34,35]. The use of surveys [16] or the application of standardized tests [23] as instruments and techniques for data collection was also highlighted.

### Selection Criteria and Participant Demographics

Sample size varied significantly in all the studies, ranging from 5 [35] to 62 [16]. Only 1 study did not specify the sample size, although it referred to older adults with reported cognitive impairment [14]. In 3 of the 6 studies, gender was specified, with predominantly female participants in 2 studies [15,23] and male participants in 1 study [16]. These 3 studies also reported the age of the participants, with an average of 69 years [15], 66 years [16], and 81 years [23]. Regarding place of residence, the home or residential centers were the norm in 3 studies [14-16], with residence in rural areas being relevant in 1 study [35]. Two studies omitted data on place of residence [23,34]. The diagnosis of an illness was an eligibility criterion in 3 studies, specifically Parkinson disease [15,16] and macular degeneration with oculomotor deficits [23]. No study detailed data on the occupation, educational level, or degree of digital literacy and technological training of the participants.

A wide variety of AI-enabled smart glasses were considered in the different studies, highlighting Microsoft HoloLens 2 [14], Google Glass [15], Epson Moverio BT-200 [23], Epson Moverio BT-300 [34], and Jorjin J7EF AR smart glasses [35]. Only 1 study addressed this type of technology in a generalized manner without specifying the model of glasses [16]. In addition to AI-enabled smart glasses, in the included studies, other technologies and devices were used, such as intelligent robots and virtual assistants [14], tablets and smartphones [16], cloud computing [34], and a portable Android device that performs real-time data processing (eg, image analysis or posture calculation) to send information to the glasses while the user moves or exercises [35].

The promotion of autonomy and the improvement of motor functions were the most noteworthy benefits as AI-enabled smart glasses can provide visual support or auditory cues by overlaying information in real time [14-16]. Similarly, benefits for physical health were evidenced through a more active lifestyle and increased mobility of the participants [15,35], as well as positive results in social interactions [14]. Another benefit identified was the improvement in reading performance and text readability due to the dynamization and personalization via AI-enabled smart glasses [23,34].

The main barriers or hindering factors that were identified in the results of the studies included sociocontextual, distinguishing privacy issues and network vulnerabilities [14] or the high cost of the devices [15,16]. In terms of the inherent factors of the technology itself, such as its design and technical characteristics, some glasses may be inconvenient to use for this sector of the population due to the reduced size of the displays or a limited audio volume [15], as well as the possible absence of blue light filters in some displays [16]. An internal barrier was the lack of evaluations of the usability and acceptance of these technologies among older adults [14].

## Discussion

### Principal Findings

Preliminary findings suggest improvements in quality of life, independence, motor function, and social relationships among older adults resulting from the use of AI-enabled smart glasses. In terms of quality of life and overall well-being, aspects such as lack of social connections, independent living, or decreased mobility of older adults are some of the current concerns in this population. AI-enabled smart glasses have emerged as digital enabling technologies to alleviate these issues.

This scoping review contributes to scientific literature by mapping the available evidence on this type of technology and identifying indicators of effectiveness and potential benefits in promoting active aging. As this is an emerging technology with a marked innovative character, there are currently very few studies on it. Therefore, the contribution of this scoping review, from a holistic point of view, is even more significant. AI-enabled smart glasses facilitated independence, functional autonomy, and improvement of motor skills in the context of active and healthy aging [14-16]. These benefits were produced because these portable devices contribute to more conscious decision-making and greater confidence in the actions performed, as well as improvements in motor performance skills such as coordination, posture, and balance. For these reasons, AI-enabled smart glasses have the potential to empower older adults in the performance of their daily tasks and routines [16,34].

In addition, the implementation of AI-enabled smart glasses in this sector of the population produced an increase in daily physical activity levels due to the ubiquitous nature of this type of technology [15,35]. In other studies, links were found between the adoption of AI-enabled smart glasses and improvements in social connections through home automation [14]. It has also been demonstrated that AI-enabled smart glasses facilitate reading in real time [34]. In this sense, these technologies could offer alternatives for entertainment and personal enjoyment [15].

It was also shown that the use of AI-enabled smart glasses minimized symptoms of diseases such as Parkinson disease or macular degeneration [15,16,23]. All this translates into a significant increase in well-being and quality of life through daily assistive devices [14,16,35].

However, some hindering factors and/or barriers were identified regarding both the adoption and implementation of these wearable devices among older adults. First, more research is needed to guarantee aspects related to the safety, effectiveness, usability, and social acceptance of AI-enabled smart glasses regarding active living in old age [16].

Second, it is important that AI-enabled smart glasses are compatible with the prescription glasses worn by older adults, are easy to use, include a simple and natural interface, and recognize broad gestures rather than overly precise keystrokes. All these features would facilitate navigation [16].

The use of displays without blue light filters in this type of technology could alter quality of sleep and circadian rhythm. In fact, prolonged use of AI-enabled smart glasses may cause a feeling of visual fatigue or mild dizziness [15].

Furthermore, the efficient use of these digital technologies still needs to address the limited digital literacy of many older people [36,37]. In other words, the lack of technological skills and basic competencies in the use of electronic devices and digital tools is a limiting factor in the adoption of these devices and autonomous and independent management [38-40], showing the need to promote training strategies for digital learning and empowerment in older adults.

### Limitations and Future Research

Although only 6 studies fulfilled the inclusion criteria following an exhaustive search that identified 58 documents, we consider that this scoping review is both relevant and valid, given that the subject of study (AI-enabled smart glasses) represents an emerging technology. Consequently, it is to be expected that scientific literature in this area remains limited or is still in the early stages of development. This review mapped the current state of knowledge and identified gaps in the literature, offering a meaningful and relevant preliminary overview that is consistent with the objectives of a scoping review. However, publication bias, methodological heterogeneity, sample size constraints, the instruments used, and the limited availability of rigorous conceptual frameworks, together with inherent language restrictions, made it difficult to establish relationships among the included studies. Therefore, further empirical research with more rigorous methods and periodic reviews is suggested as the scientific output grows and new AI-enabled smart glasses become commercially available.

### Conclusions

The available studies included in this scoping review suggest that AI-enabled smart glasses enable improvements in the quality of life of older adults, especially in capabilities and skills such as independence, autonomy, and motor function. Preliminary evidence also indicates higher levels of physical activity and increased social interactions through the use of AI-enabled smart glasses. Although these portable devices could offer a more inclusive and accessible digital future by potentially delaying the effects of aging and contributing to active living in old age, these results should be interpreted cautiously given the limited number of studies and methodological heterogeneity.

This scoping review used a synthesis of evidence to clarify the state of the art and expand existing knowledge on the role of these digital technologies in promoting active aging, in addition to identifying gaps in research that serve as a basis for future studies. It is necessary to explore more deeply the potential mechanisms underlying aging delay through this type of digital technology, which requires research with larger samples and longitudinal designs. Moreover, successful implementation and adoption require addressing digital literacy and ongoing training in technological skills among this population segment, as well as addressing challenges related to data protection, usability, acceptance, efficiency, and technical complexity.

## Supplementary material

10.2196/81157 Multimedia Appendix 1 Organization of data as a synthesis of the final included studies and their key findings.

10.2196/81157 Checklist 1 PRISMA-ScR checklist.

## Abbreviations

AI: artificial intelligence
PRISMA: Preferred Reporting Items for Systematic Reviews and Meta-Analyses
PRISMA-ScR: Preferred Reporting Items for Systematic Reviews and Meta-Analyses extension for Scoping Reviews
