# Supplementary material for: AI-Enabled Smart Glasses for Active Aging: Scoping Review
Source: JMIR Aging. 2026 Feb 25;9:e81157. doi: 10.2196/81157 (PMC12935290; doi:10.2196/81157)
Supplement: Multimedia Appendix 1 [file aging-v9-e81157-s001.docx]

**Multimedia Appendix 1.** Organization of data as a synthesis of final included studies and key findings.

| Author and year of publication | Title | Type of document | Country of origin | Objective | Methodology | Sample | AI-enabled smart glasses used and/or analyzed | Main results |
| --- | --- | --- | --- | --- | --- | --- | --- | --- |
| [1] | A^2^Fitness: An Artificial Intelligent Fitness Assistance System Using Augmented Reality Smart Glasses for Elderly Health-Promotion | Conference paper | Taiwan | Demonstrate the effectiveness of an assistance system for fitness improvement in older adults, called A^2^Fitness, composed of AI and augmented reality (AR) technology subsystems | Quantitative preliminary study. Participants performed three fitness exercises at least once a day for three months. The accuracy of the system was evaluated, and the recovery rate and number of repetitions were measured before (pretest) and after (posttest) performing the exercises | 5 older adults | J7EF AR smart glasses | The results reveal that A^2^Fitness is a very suitable tool to effectively promote the health of older adults, obtaining a significant improvement in the number of fitness exercises in all participants, given that they can use AI-enabled smart glasses anytime, anywhere. The proposed assistance system can achieve a high degree of recovery rate regardless of the physical exercise performed by the elderly |
| [2] | Dynamic text presentation on smart glasses: A pilot evaluation in age-related macular degeneration | Article | United Kingdom | Test the efficacy of AI-enabled smart glasses to facilitate the reading process in older adults with low vision by categorizing eye movements during reading | Quantitative. Pilot study through which an assessment of reading speed was performed and a questionnaire was administered in order to measure the subjective preferences and self-assessment of the participants, comparing reading speed with AI-enabled smart glasses with the reading of printed text on paper using the usual optical aid | 23 older adults with a diagnosis of macular disease and oculomotor deficits affecting reading ability | Epson Moverio BT200 | The results provide initial evidence of the effectiveness of AI-enabled smart glasses with respect to an increase in reading speed in older adults with macular disease, as the majority of participants read faster with personalized text in AI-enabled smart glasses |
| [3] | Social Embodiment of Companion Robots in Smart Spaces: IoRT for Independent Living | Conference paper | Canada | Integrate personal smart devices and home automation technologies with social companion robots and AI-enabled smart glasses to assist with daily tasks, efficiently manage the home environment, and support the emotional well-being of older adults through affective computing | Quantitative. Integration of a Social Companion Robot (SCR), together with AI-enabled smart glasses, in homes and residences of older adults requiring full or partial care. Quantitative experiments were conducted on facial emotion recognition in the framework of affective computing, as well as metrics regarding the performance of the devices used | Older adults with self-reported cognitive impairment. Sample size not specified | Microsoft Hololens 2 | The implementation of SCRs can improve the quality of life of older adults in relation to their independence, autonomy and social interactions, as well as control intelligence, with more context- and situation-aware decision making |
| [4] | Augmented reality-based dance intervention for individuals with  Parkinson’s disease: A pilot study | Article | New York | Assess the feasibility, acceptability, and safety of AI-enabled smart glasses use in older adults with Parkinson's disease through dance-centered physical activity adherence | Mixed. Feasibility pilot study of only one group. After confirming eligibility, participants completed a semi-structured intake interview, a set of questionnaires, and baseline motor function assessments. Subsequently they received a 20-minute demonstration on the use of AI-enabled smart glasses. Three weeks later, the motor function assessments were repeated with a second set of questionnaires and an exit interview | 7 older adults with Parkinson’s disease | Google Glass | The domestic use of AI-enabled smart glasses to promote adherence to physical activity is feasible in people with Parkinson’s disease. The participants reported high degrees of satisfaction and acceptability, with improvements in motor functions |
| [5] | Development of an Assistive Device via Smart Glasses | Conference paper | Taiwan | Develop and assess an assistive device, aimed at older adults, using AI-enabled smart glasses with facial recognition functions with memory retrieval, image enlargement and optimization, and control through voice commands | Quantitative. Three rounds of facial recognition tests were carried out using machine learning algorithms. AI-enabled smart glasses were used to capture images in real time and calculate facial features in order to obtain a person’s ID. Furthermore, a cloud database was integrated with the intention of managing the information associated with the different faces. Also, image processing and binarization were used to improve readability, as well as voice commands using Google recognition | 50 older adults | Epson Moverio BT-300 | Increased accuracy of facial recognition and better readability of text through increased contrast. Likewise, five functional voice commands were implemented for the basic control of the device |
| [6] | E-health Support in People with Parkinson’s Disease with Smart Glasses: A Survey of User Requirements and Expectations in the Netherlands | Article | The Netherlands | Assess the requirements, limitations and attitudes of older adults with Parkinson’s disease regarding the use and adoption of AI-enabled smart glasses | Quantitative. Online survey to find out opinions on AI-enabled smart glasses and how these devices can contribute to the quality of life of older adults | 62 older adults with Parkinson’s disease | AI-enabled smart glasses in general | The participants were receptive to AI-enabled smart glasses as assistive technology to facilitate daily activities, especially due to their potential to self-manage motor problems and provide guidance, improving levels of self-confidence and independence. The reported use suggests that AI-enabled smart glasses could be adopted relatively easily. This type of technology could even predict when the person would need assistance and take precautionary measures depending on the situation, in addition to complementing existing therapies for Parkinson's disease |

**References**

1. Chang WJ, Chen HW, Su JP, et al. A^2^Fitness: An artificial intelligent fitness assistance system using a augmented reality smart glasses for elderly health-promotion. 2023 International Conference on Consumer Electronics-Taiwan (ICCE-Taiwan); 2023 Jul 17-19; PingTung, Taiwan. Piscataway, NJ: IEEE; 2023. doi: 10.1109/ICCE-Taiwan58799.2023.10227060
2. Moshtael H, Tooth C, Nuthmann A, et al. Dynamic text presentation on smart glasses: A pilot evaluation in age-related macular degeneration. Br J Vis Impair. 2019 Dec;38(1):24-37. doi: 10.1177/0264619619889998
3. Muema C, Lawrence S, Anjum T, et al. Social embodiment of companion robots in smart spaces: IoRT for independent living. In: Galambos P, Kayacan E, Madani K, editors. Robotics, computer vision and intelligent systems. 1st ed. Cham, Switzerland: Springer; 2022:147-171. ISBN: 9783031196492
4. Tunur T, DeBlois A, Yates-Horton E, et al. Augmented reality-based dance intervention for individuals with Parkinson’s disease: A pilot study. Disabil Health J. 2020 Oct;13(2):100848. PMID: 31679951
5. Wang C-S, Huang W, Chang Y-F, et al. Development of an assistive device via smart glasses. 2020 IEEE 2nd Eurasia Conference on Biomedical Engineering, Healthcare and Sustainability (ECBIOS); 2020 May 29-31; Tainan, Taiwan. Piscataway, NJ: IEEE; 2020. doi: 10.1109/ECBIOS50299.2020.9203629
6. Zhao Y, Heida T, van Wegen EEH, et al. E-health support in people with Parkinson’s disease with smart glasses: A survey of user requirements and expectations in the Netherlands. J Parkinsons Dis. 2015 Apr;5(2):369-378. PMID: 25855044
